# The central amygdala modulates distinctive conflict-like behaviors in a naturalistic foraging task

**DOI:** 10.3389/fnbeh.2023.1212884

**Published:** 2023-08-02

**Authors:** Sunwhi Kimm, Jeansok J. Kim, June-Seek Choi

**Affiliations:** ^1^School of Psychology, Korea University, Seoul, Republic of Korea; ^2^Department of Psychology, University of Washington, Seattle, WA, United States

**Keywords:** conflict, predator, defensive behavior, amygdala, foraging

## Abstract

Conflict situations elicit a diverse range of behaviors that extend beyond the simplistic approach or avoidance dichotomy. However, many conflict-related studies have primarily focused on approach suppression, neglecting the complexity of these behaviors. In our study, we exposed rats to a semi-naturalistic foraging task, presenting them with a trade-off between a food reward and a predatory threat posed by a robotic agent. We observed that rats displayed two conflict-like behaviors (CLBs)—diagonal approach and stretched posture—when facing a robotic predator guarding a food pellet. After electrolytic lesions to the central amygdala (CeA), both conflict behaviors were significantly reduced, accompanied by a decrease in avoidance behavior (hiding) and an increase in approach behavior (frequency of interactions with the robot). A significant negative correlation between avoidance and approach behaviors emerged after the CeA lesion; however, our data suggest that CLBs are not tightly coupled with either approach or avoidance behaviors, showing no significant correlation to those behaviors. Our findings indicate that the CeA plays a crucial role in modulating conflict behaviors, competing with approach suppression in risky situations.

## Introduction

Conflict behaviors are frequently observed in nature as animals strive to navigate the complex interplay between potential rewards and risks. Foraging animals, for example, must continuously weigh the benefits of obtaining food against the potential threats of predation [e.g., (Lima and Dill, 1990)]. This behavior can be described as a combination of approach and avoidance strategies. Researchers have identified specific conflict-like behaviors (CLBs) such as moving back and forth (Miller, 1944), head dips (Takeda et al., 1998), and stretched postures (Mackintosh and Grant, 1963; Kaesermann, 1986; Blanchard and Blanchard, 1989), which may be regulated by distinct mechanisms separate from approach or avoidance (Gray, 1977; Corr, 2013; McNaughton and Corr, 2014). However, most conflict studies have focused on the dichotomy between avoidance and approach suppression, assuming that CLBs are highly correlated with and adequately represented by these behavioral indexes. For example, commonly used conflict tests include the probabilistic administration of electric foot shocks and the measurement of suppressed licking (Vogel et al., 1971; Millan and Brocco, 2003; Burgos-Robles et al., 2017; Schumacher et al., 2018; Choi et al., 2019) or conditioned lever-pressing (Piantadosi et al., 2017). Other studies have focused on withholding or delaying approach to circumvent aversive outcomes (Bravo-Rivera et al., 2014) or opting for less rewarding behavioral choices over more risky and aversive situations (Friedman et al., 2015).

In this study, we aimed to observe the emergence of defensive behaviors in foraging rats by using a semi-naturalistic foraging task. To achieve this, we utilized Lobsterbot, a robotic agent designed to guard a food pellet and threaten approaching rats with a snapping motion (Kimm and Choi, 2018). Previous research has demonstrated that robotic predators can elicit realistic and effective threat responses in laboratory rats (Choi and Kim, 2010; Amir et al., 2015; Kim et al., 2018; Kimm and Choi, 2018; Lee et al., 2018). Our experimental setup allowed us to identify two distinct CLBs in rats: diagonal approach and stretched posture. A diagonal approach is characterized by a cautious trajectory along the walls toward the goal, in contrast to the direct approach through open space typically observed in goal-directed navigation without threats. In a stretched posture, rats exhibit a distinct body profile, with an elongated torso, hind paws further from the body’s center, and front paws gradually approaching the goal.

We further investigated the effect of lesions in the central nucleus of the amygdala (CeA) on CLBs, as previous research has associated the CeA with conflict behaviors through indirect measures such as punished drinking (Möller et al., 1997; Taksande et al., 2014). Our findings revealed that both diagonal approach and stretched posture were reduced following CeA lesions, thereby confirming the CeA’s crucial role in regulating innate CLBs.

## Methods

### Subjects

Male Sprague-Dawley rats (Orient Bio, Kyunggi-do, Republic of Korea), initially weighing 250–270 g, were used. All animals were individually housed in a climate-controlled vivarium with a reverse 12-h light/dark cycle (lights on at 9:00 PM). Experiments were conducted during the dark phase of the cycle and strictly followed the guidelines for the “Care and Use of Laboratory Rats” from Korea University, Seoul, Republic of Korea.

### Surgery

Rats were anesthetized with pentobarbital sodium (50 mg/kg, i.p.) and mounted on a stereotaxic frame. Two holes were drilled into the exposed cranium, and custom-made electrodes (stainless steel insect pins insulated with epoxy, except for 0.5–1 mm at the tip) were inserted through these holes to reach the target coordinates in the CeA. Bilateral lesions in the CeA were made by passing anodal currents, with coordinates and lesion parameters detailed in Supplementary Table 1 (Paxinos and Watson, 2006). Rats in the sham lesion group (SHAM) underwent the same surgical procedure except that the electrodes were inserted 1–2 mm dorsal to the target coordinates, and no current was passed. All rats were given a recovery period of 7–14 days after the surgery was completed and placed on a standard food deprivation schedule to maintain 80–90% of their normal body weights.

### Lobsterbot task

**FIGURE 1:**
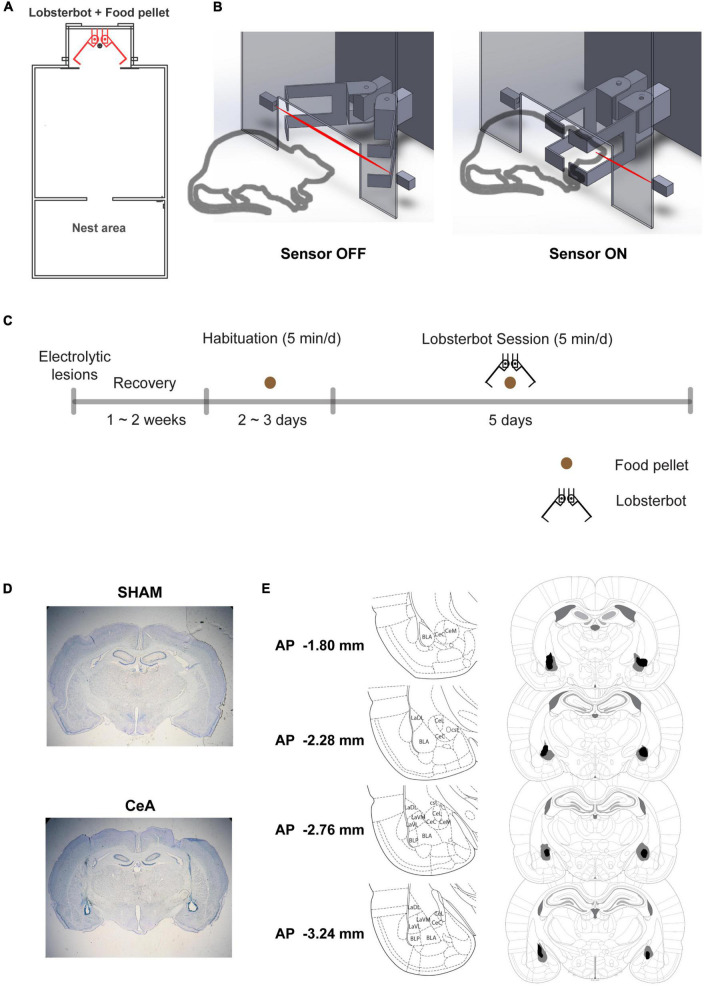
Experimental setup and procedure. **(A)** Schematic of the experimental arena, highlighting the positions of the Lobsterbot, food pellet, and Nest area. **(B)** Diagram of a rat-Lobsterbot interaction. The Lobsterbot’s claws remain open at rest but close with a velocity of 164 ms/40° when the rat’s head breaks the infrared photograph beam. **(C)** Overview of the experimental design. First, bilateral electrolytic lesions or sham lesions were applied to the central amygdala of rats. After a 2-week recovery period, the rats were habituated to the arena with just the food pellet and no Lobsterbot present. Habituation ended after two consecutive sessions in which the rats consumed the pellets. Finally, five Lobsterbot sessions were conducted where the Lobsterbot guarded the food pellet. **(D)** Photomicrographs showing representative SHAM and CeA lesions. **(E)** Reconstruction of CeA lesions, with dark regions indicating the least extensive lesions and gray regions indicating the most extensive lesions. The distances from bregma are indicated in the leftmost column, and the coordinates were adapted from the rat brain atlas by Paxinos and Watson (6th edition).

All rats underwent an approach-avoidance conflict task that had been previously developed (Kimm and Choi, 2018). The task involved a robot named Lobsterbot (Figure 1A) designed to mimic the prey-capturing motion of a predator, obstructing the rat’s access to a food pellet (1.5–2 g) by snapping its claws (Figure 1B).

The experimental protocol consisted of habituation and Lobsterbot sessions (Figure 1C). First, the rats were habituated to the experimental arena for 2–3 days without the Lobsterbot present. During this time, the rats were allowed to freely explore the arena for 5 min, and a single food pellet was available at the opposite end of the arena. The habituation stage for each rat ended when they consumed food pellets in 2 consecutive sessions.

After completing the habituation stage, the Lobsterbot sessions (5 min/day) began and lasted for 5 days. During these sessions, the Lobsterbot was positioned behind the food pellet and snapped its claws when it detected the rat’s presence. A breach in the infrared photobeam was considered an episode of encounter, and the Lobsterbot’s attack ended as soon as the rat escaped and the photobeam was no longer broken.

### Histology

After the completion of the experiments, rats were overdosed with sodium pentobarbital and perfused transcardially with saline and 10% buffered formalin. The brains were stored in post-fix solution (30% sucrose in 10% buffered formalin) and sectioned on a microtome at 50 μm thickness. Sections were mounted on gelatin-coated slides, then stained with Cresyl violet and Prussian blue dyes. The brain lesion sites were histologically reconstructed for the analysis (Figures 1D, E).

### Behavioral measurement

**FIGURE 2:**
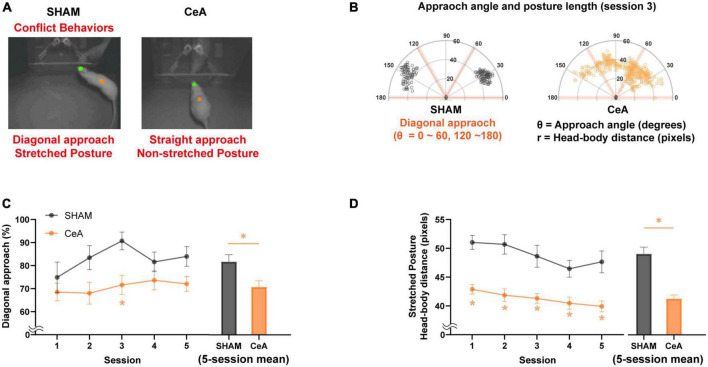
Impact of CeA lesions on conflict behaviors. **(A)** Representative images showing the difference in conflict-like behaviors between sham-lesioned (left) and CeA-lesioned (right) rats while approaching food/Lobsterbot. The green and orange dots represent the positions of the rat’s head and center of the body, respectively. While the SHAM rats typically showed a diagonal angle of approach with stretched posture, the CeA-lesioned rats showed a non-diagonal approach with non-stretched posture. **(B)** Polar plots illustrating the approach angles and posture lengths of rats from each group during their approach to food/Lobsterbot. A diagonal approach angle was defined as being between 0 and 60° or 120 and 180°. **(C)** Mean percentage of diagonal approach observed in each session (left) and across all sessions (right). **(D)** Mean length of stretched posture observed in each session (left) and across all sessions (right). Statistically significant differences between groups are indicated by an asterisk (*p < 0.05). Data are presented as mean ± SEM.

**FIGURE 3:**
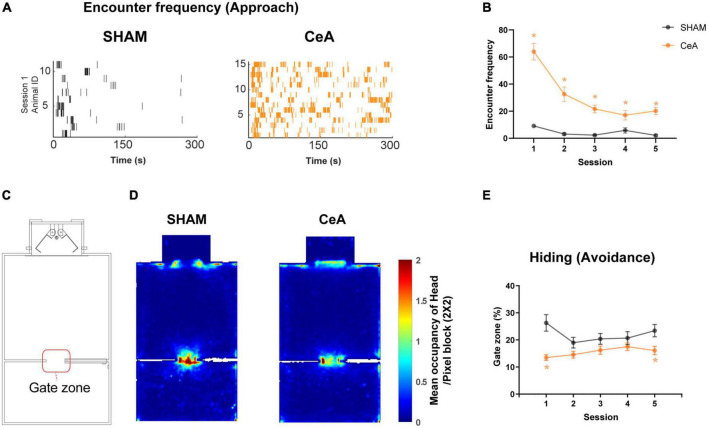
Impact of CeA lesions on approach and avoidance behaviors. **(A)** Raster plots showing robot encounters on the first Lobsterbot session. Each tick represents an episode of an encounter, and each row of ticks indicates all encounters recorded from a single rat. **(B)** Mean frequency of encounters in each session. **(C)** Map of the arena showing the location of the Gate zone. **(D)** Representative heat maps showing averaged occupancy plots over all sessions superimposed over the map of the arena. A warmer color indicates higher occupancy. **(E)** Mean duration of time spent in the Gate zone on each session. Statistically significant differences between groups are indicated by an asterisk (*p < 0.05). Data are presented as mean ± SEM.

The movements of the rats were tracked using ANY-maze video tracking software (Stoelting Co., USA). The software tracked the green dot, which represented the rat’s head, and the orange dot, which represented the center of the rat’s body. These movements were then used to calculate the angle and length of the stretched posture during the rat’s encounter with the Lobsterbot (as shown in Figures 2A, B). The frequency of encounters was monitored using the infrared photobeam sensors of the Lobsterbot (as shown in Figure 3A). Additionally, ANY-maze also monitored the position and movement of the rats to measure the time they spent hiding in the Gate zone (an area around the gate, as shown in Figures 3C, D).

### Statistical analysis

The statistical analysis was conducted using IBM SPSS version 20.0. A two-way ANOVA with repeated measures was applied to examine behaviors (diagonal approach, stretched posture, encounter frequency, and duration in the Gate zone) for each session. Bonferroni’s *post-hoc* tests were conducted to compare both between-group and within-group differences in all the aforementioned behaviors. An independent *t*-test was utilized to evaluate the between-group difference in the mean percentage of diagonal approach and stretched posture across five sessions. Pearson’s correlation coefficient was employed to analyze the relationship between behavioral indices. Results are presented as the mean ± standard error of the mean (SEM).

## Results

### Effects of CeA lesions on conflict-like behaviors (CLBs)

SHAM (*n* = 11) and CeA-lesioned (*n* = 15) rats were tested in an approach-avoidance conflict task using the Lobsterbot. During the task, rats foraged for food pellets while a predator-like robot, Lobsterbot, obstructed access to the pellets by snapping its claws (Figure 1B and Supplementary Video 1). The rat’s head and body center were tracked to determine the approach angle and stretched posture (Figures 2A, B).

The results showed that most rats in the SHAM group favored a diagonal approach angle (θ < 60°, 120° θ < 180°) over a straight approach angle (60° < θ < 120°) when approaching the Lobsterbot. The mean percentage of diagonal approaches was significantly affected by the lesion [Two-way repeated measures ANOVA; *F*_(1,23)_ = 8.336, *p* < 0.01; Figure 2C-left], but not by session [*F*_(3.206,73.73)_ = 1.671, *p* > 0.1] or lesion × session interaction [*F*_(4,92)_ = 0.6426, *p* > 0.6]. CeA-lesioned rats showed a significantly lower percentages of diagonal approaches compared to the SHAM rats in the third session (Bonferroni’s *post hoc*, *p* < 0.05; Figure 2C-left). On average, the CeA-lesioned rats displayed a significantly lower percentage of diagonal approaches over the course of 5 sessions compared to the SHAM rats [Independent *t*-test; *t*_(24)_ = 2.656, *p* < 0.05; Figure 2C-right].

The stretched posture analysis revealed significant main effects of lesion [Two-way repeated measures ANOVA; *F*_(1,23)_ = 32.87, *p* < 0.0001; Figure 2D-left] and session [*F*_(3.184,73.23)_ = 5.282, *p* < 0.01], but not of lesion × session interaction [*F*_(4,92)_ = 0.6426, *p* > 0.6]. CeA-lesioned rats exhibited significantly shorter stretched postures compared to the SHAM rats across all sessions (Bonferroni’s *post hoc*, *p*-values < 0.05; Figure 2D-left) as well as in the 5-session average [Independent *t*-test; *t*_(24)_ = 6.117, *p* < 0.05; Figure 2D-right].

To sum, both diagonal approach and stretched posture were reduced by CeA lesions, indicating that CeA is critical for the expression of CLBs.

### Alterations in approach and avoidance behaviors by CeA lesions

In addition to the reduced CLBs, CeA lesions also altered the approach and avoidance. The frequency of encounters, used as an index of approach, was significantly increased by the lesion (Figures 3A, B). A Two-way repeated measures ANOVA revealed significant effects of lesion [*F*_(1,24)_ = 50.30, *p* < 0.0001], session [*F*_(2.631,63.15)_ = 30.11, *p* < 0.0001], and interaction [*F*_(4,96)_ = 18.73, *p* < 0.0001] (Figure 3B). *Post hoc* analysis further indicated that the frequency of encounters in the CeA-lesioned rats was significantly higher than in the SHAM-lesioned rats across all sessions (Bonferroni’s *post hoc*, *p*-values < 0.05; Figure 3B).

Avoidance was assessed by the time spent hiding in the Gate zone (a virtual 12 cm × 12 cm zone depicted in Figure 3C). The increased hiding time was observed when the rats avoided the Lobsterbot and preferred a location behind the walls in the Gate zone, as reported in a previous study (Kimm and Choi, 2018). This behavior is best characterized as ‘inhibitory avoidance’, which involves suppressing approach behaviors in the presence of the Lobsterbot. It represents a more passive reaction to threat, differing from active forms of avoidance or escape. CeA lesions significantly decreased the time spent hiding (Figures 3D, E). A Two-way repeated measures ANOVA found a significant effect of the lesion [*F*_(1,24)_ = 13.12, *p* < 0.0014] (Figure 3E). *Post-hoc* analysis also showed that the hiding time was lower in CeA-lesioned rats in sessions 1 and 5 (Bonferroni’s *post hoc*, *p*-values < 0.05; Figure 3E). Taken together, these results suggest that the CeA is critical for approach suppression and threat avoidance. Likewise, the pattern of time spent in the Nest area mirrored that of the Gate zone, even though the duration in the Nest area was approximately twice as long as in the Gate zone for both groups (Supplementary Figure 1).

Additionally, head-withdrawal, an active escape behavior from the Lobsterbot was measured (Supplementary Figure 2). The head-withdrawal latency measured at milliseconds scale was significantly slowed by the CeA lesion across all sessions, indicating CeA’s role in reflexive defensive behavior as well.

### Correlational analysis of conflict, approach, and avoidance behaviors

**FIGURE 4:**
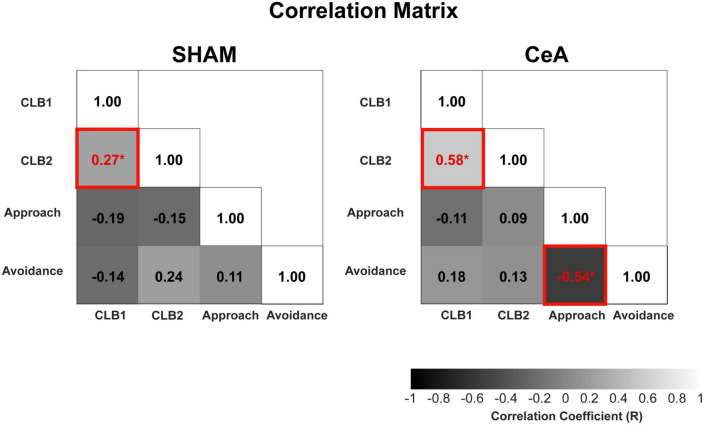
Correlational analysis of conflict, approach, and avoidance behaviors. Correlation matrices of SHAM and CeA-lesioned groups. The matrices show correlations between CLBs (CLB1: diagonal approach, CLB2: stretched posture), approach behavior (encounter frequency), and avoidance behavior (hiding in the Gate zone). The numbers in the matrices represent the correlation coefficients. Increased brightness indicates higher coefficients, and asterisks (*) indicate statistically significant coefficients (*p* < 0.05).

The significant impact of CeA lesions on CLBs and approach/avoidance behaviors raises the question of whether the decreased conflict behaviors are simply byproducts of reduced avoidance. To address this question, a correlational analysis was conducted among all behaviors (Figure 4). In the SHAM-lesioned rats, the diagonal approach showed a significant and positive correlation with the stretched posture (*r* = 0.27, *p* < 0.0001). These two conflict behaviors were not significantly correlated with any other behavioral indices of approach or avoidance, such as encounter frequency or hiding (*p*-values > 0.05). In the CeA-lesioned rats, the correlation between the two conflict behaviors remained robust (*r* = 0.58, *p* < 0.0001), without any significant correlation to other approach and avoidance behaviors (*p*-values > 0.05). These results suggest that the two conflict behaviors are not directly tied to approach suppression or avoidance behaviors and that CeA lesions have a direct impact on CLBs rather than an indirect influence through those behaviors. Interestingly, the correlation between the frequency of encounter (approach) and time spent hiding (avoidance), which was not significant in SHAM, became significantly negative in the CeA lesion (*r* = −0.54, *p* < 0.0001), suggesting that the inverse relationship between approach and avoidance becomes prominent when the CeA no longer dominantly regulates those behaviors.

## Discussion

Despite an ample number of studies investigating approach and avoidance behaviors in conflict situations, only a few have focused on identifying CLBs and the related neural circuits. CLBs in rodents might not have been frequently identified in most laboratory settings, perhaps because they appear more prominently in naturalistic foraging environments where a variety of behavioral modules need to be recruited for optimal performance (Miller, 1944; Mackintosh and Grant, 1963; Gray, 1977; Kaesermann, 1986; Blanchard and Blanchard, 1989; Takeda et al., 1998). There has been a need for novel behavioral paradigms to induce CLBs in laboratory settings because the majority of studies have defined conflict as avoidance (Shibata et al., 1986; Yamashita et al., 1989) or delayed approach (Bravo-Rivera et al., 2014), rather than directly examining CLBs. A more recent study developed a battery of conflict tests to better assess CLBs in rodents (Illescas-Huerta et al., 2021). Specifically, these tests employed tasks consisting of various challenges, for example, a crossing-mediated conflict task where rats had to cross a potentially electrified grid to obtain food at the opposite end of a straight alley. Several CLBs were identified, including hesitations (touching the threat zone then aborting the approach), stretched posture, and head-dipping. It is important to note that CLBs often manifest as approach behaviors despite the presence of threats. Therefore, simply counting the reduction in the number of approaches may not be a valid representation of conflict.

In the current study, we employed an innate conflict task to observe frequent expressions of CLBs. In a realistic foraging task, rodents faced a constant threat from an automated, sensor-activated agent while attempting to access a visibly attainable food reward. This setup allowed us to observe the emergence of CLBs, such as diagonal approach and stretched posture, without the need for training or conditioning trials. Additionally, these CLBs were not correlated with approach or avoidance behaviors, indicating that they may be a separate class of behaviors. Importantly, the observed CLBs differ from displacement behaviors, which typically occur during stressful situations and often involve unrelated or out-of-context actions, such as grooming or scratching (Maestripieri et al., 1992; Spruijt et al., 1992). In contrast, CLBs observed in our study appear to be more directly related to the conflict situation, reflecting the animal’s attempt to balance approach and avoidance in the presence of a threat.

Furthermore, we investigated the CeA’s role in CLBs. Our data showed that CeA lesions led to a significant reduction in the display of CLBs, alongside even more substantial changes in approach and avoidance behaviors. This underscores the CeA’s involvement in all three behaviors–approach, avoidance, and CLBs. While previous studies have suggested the CeA’s role in approach and avoidance (Knapska et al., 2006; Lázaro-Muñoz et al., 2010; Schlund and Cataldo, 2010; Kim et al., 2017), it remained unclear whether the impact of CeA lesions on CLBs was a direct effect or a secondary consequence of alterations in approach or avoidance behaviors. Previous studies investigating the effect of CeA lesions in conflict behaviors measured disinhibition of approach as an index of reduced conflict, such as increase in punished drinking (Yamashita et al., 1989; Möller et al., 1997). This led to critiques regarding the construct validity of the findings. Indeed, the CeA has been implicated in defensive behaviors like freezing (LeDoux et al., 1988; Choi and Brown, 2003), and the disinhibition of approach could have likely resulted from reduced defensive behavior. In contrast, our findings suggest that the CeA may have direct impact on CLBs that is less closely related to approach and avoidance behaviors. The changes in CLBs induced by CeA lesions showed no significant correlation to the changes in approach/avoidance behaviors, whereas a significant negative correlation between approach and avoidance behaviors was found. This supports the idea that the CeA may control conflict behaviors directly to some level, rather than merely as a byproduct of approach or avoidance behaviors.

In conclusion, the present study offers novel insights into the role of the central nucleus of the amygdala (CeA) in regulating approach-avoidance conflict (AAC) and conflict-like behaviors (CLBs) in rats, employing an ethologically valid, semi-natural setting that allows for the observation of these behaviors distinct from approach and avoidance. Our findings emphasize the significance of the CeA in governing both CLBs and avoidance behaviors, illustrating that the underlying mechanisms for these behaviors within the CeA are likely distinct. Future research adopting this methodology and finer control over sub-populations of neurons within the CeA could enhance our understanding of the neural mechanisms underlying conflict, explore the precise neural mechanisms within the CeA that control CLBs, and further investigate the relationship between the CeA and other brain regions involved in conflict regulation, thereby reinforcing the validity of the findings.

## Data availability statement

The raw data supporting the conclusions of this article will be made available by the authors, without undue reservation.

## Ethics statement

The animal study was reviewed and approved by the Institutional Animal Care and Use Committee of Korea University.

## Author contributions

J-SC and SK conceived the key ideas and designed the experiment. SK collected the data. J-SC, SK, and JJK analyzed the data and wrote the manuscript. All authors contributed to the article and approved the submitted version.
