# Supplementary material for: The central amygdala modulates distinctive conflict-like behaviors in a naturalistic foraging task
Source: Front Behav Neurosci. 2023 Aug 2;17:1212884. doi: 10.3389/fnbeh.2023.1212884 (PMC10433198; doi:10.3389/fnbeh.2023.1212884)
Supplement: Supplementary file 1 [file Data_Sheet_1.pdf]

Supplementary Material

The central amygdala modulates distinctive conflict-like behaviors in a naturalistic foraging task

Sunwhi Kimm[1], Jeansok J Kim[2] and June-Seek Choi[1]*

*** Correspondence:**

Corresponding Author:

Dr. June-Seek Choi: j-schoi@korea.ac.kr

1 Supplementary Figures and Tables

	Uninsulated tip	Anterior/Posterior	Medial/Lateral	Ventral	Current	Duration (s)
**CeA**	0.5 mm	-2.2	±4.0	-8.0, -8.5	1.0 mA	10
		-2.8	±4.3	-8.0	1.0 mA	10

Supplementary Table 1. Coordinates and parameters of electrolytic lesion

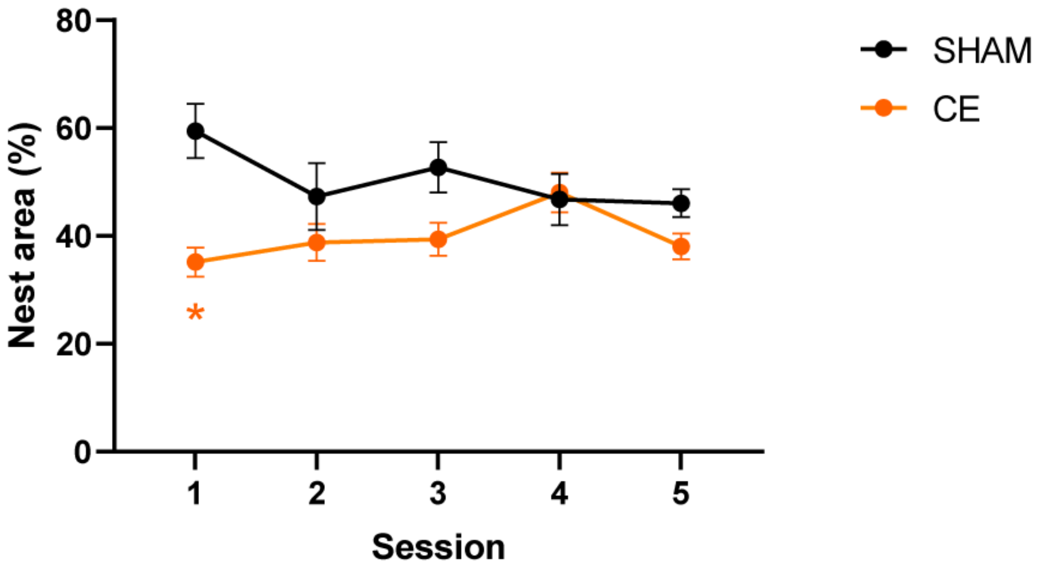

Supplementary Figure 1. Time spent in Nest area

Time spent in Nest area. An asterisk (*) indicate statistically significant difference between groups ($p < 0.05$).

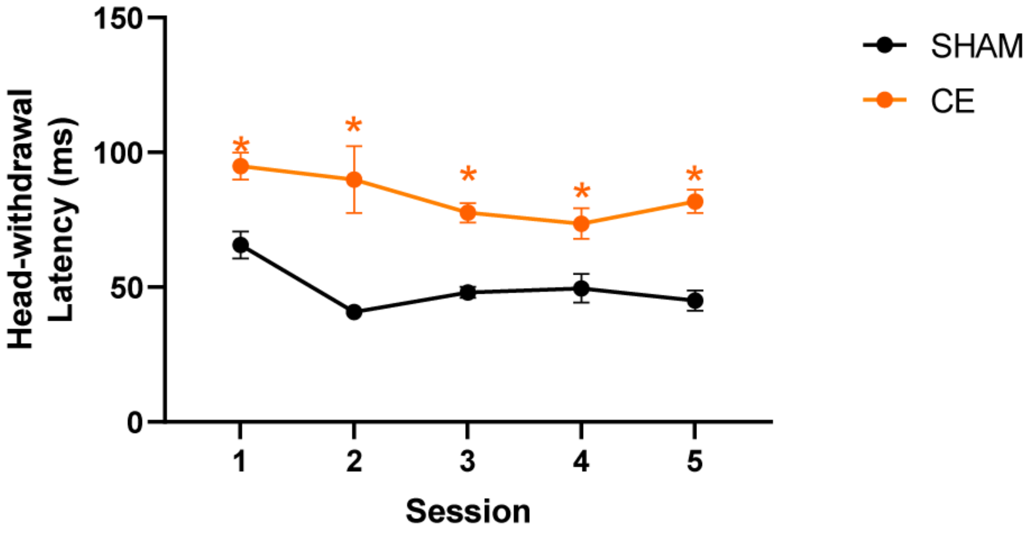

Supplementary Figure 2. Head-withdrawal latency

Latency to withdraw head from the onset of the Lobsterbot's attack. Timescale is at milliseconds. An asterisk (*) indicate statistically significant difference between groups ($p < 0.05$).

Supplementary Video 1. Lobsterbot task and effects of central amygdala lesion

URL: https://youtu.be/xdPYV0XKYRk
